# PW01-002 – Colchicine resistant FMF in Turkish children

**DOI:** 10.1186/1546-0096-11-S1-A55

**Published:** 2013-11-08

**Authors:** FK Kara Eroglu, Y Bilginer, F Ozaltin, R Topaloglu, S Ozen

**Affiliations:** 1 Pediatric Rheumatology Department, Hacettepe University Faculty of Medicine, Ankara, Turkey

## Introduction

At least 5% of familial Mediterranean fever(FMF) patients do not respond to colchicine. We present our initial treatment results with colchicine resistant patients.

## Case report

### Methods

FMF resistance was defined as having ≥2 attacks in a month and persistently high CRP and SAA levels during the attack free period, in spite of adequate colchine dose. All patients were homozygous or compound heterozygotes for MEFV mutations. All continued colchicine tratment at a mean dose of 0,04±0,01 mg/kg.

### Results

Eleven patients with mean age of 12,7±7,7 years (median 14, ranging 1,5-23 years) were studied. These patients were on colchicine treatment for a mean of 5,5±4,2 years. In one patient initially etanercept was used however, this was switched to anakinra since there was no repsonse to anti TNF treatment. A total of 7 patiets were started anakinra, however, 2 had local reactions and 2 was unresponsive; they were switched to canakinumab treatment and they all responded with normal acute phase reactants. At this time a total of 8 patients are now being treated with canakinumab with a mean duration of 10,8 ±6,8 months and 3 patients with anakinra with a mean duration of 19,6 months. One patient who is on anakinra treatment has HIDS mutation as well. There were no side effects.

## Discussion

Anti IL1 treatment is beneficial in FMF patients who are resistant to colchicine and can be used safely.

## Disclosure of interest

None declared

